# Combined approaches, including long-read sequencing, address the diagnostic challenge of *HYDIN* in primary ciliary dyskinesia

**DOI:** 10.1038/s41431-024-01599-7

**Published:** 2024-04-11

**Authors:** Andrew Fleming, Miranda Galey, Lizi Briggs, Matthew Edwards, Claire Hogg, Shibu John, Sam Wilkinson, Ellie Quinn, Ranjit Rai, Tom Burgoyne, Andy Rogers, Mitali P. Patel, Paul Griffin, Steven Muller, Siobhan B. Carr, Michael R. Loebinger, Jane S. Lucas, Anand Shah, Ricardo Jose, Hannah M. Mitchison, Amelia Shoemark, Danny E. Miller, Deborah J. Morris-Rosendahl

**Affiliations:** 1 Clinical Genetics and Genomics Laboratory, Royal Brompton and Harefield Hospitals, Guy’s and St. Thomas’ NHS Foundation Trust, London, SW3 6NP UK; 2 Division of Genetic Medicine, Department of Pediatrics, University of Washington and Seattle Children’s Hospital, Seattle, WA USA; 3 Department of Laboratory Medicine and Pathology, University of Washington and Seattle Children’s Hospital, Seattle, WA 98105 USA; 4 Primary Ciliary Dyskinesia Centre, Royal Brompton and Harefield Clinical Group, Guy’s and St. Thomas’ NHS Foundation Trust, London, SW3 6NP UK; 5 National Heart and Lung Institute, Imperial College London, London, SW3 6LY UK; 6 Genetics and Genomic Medicine Department, University College London, UCL Great Ormond Street Institute of Child Health, London, WC1N 1EH UK; 7 MRC Prion Unit at UCL, Institute of Prion Diseases, UCL, London, W1W 7FF UK; 8 Primary Ciliary Dyskinesia Centre, University Hospital Southampton NHS Foundation Trust, Southampton, SO16 6YD UK; 9 Clinical and Experimental Sciences Academic Unit, University of Southampton Faculty of Medicine, Southampton, SO16 6YD UK; 10 MRC Centre of Global Infectious Disease Analysis, Department of Infectious Disease Epidemiology, School of Public Health, Imperial College London, London, W2 1PG UK; 11 Respiratory Research Group, Molecular and Cellular Medicine, University of Dundee, Dundee, DD1 9SY UK; 12 Brotman Baty Institute for Precision Medicine, University of Washington, Seattle, WA 98195 USA

**Keywords:** Genetic testing, Genetics research

## Abstract

Primary ciliary dyskinesia (PCD), a disorder of the motile cilia, is now recognised as an underdiagnosed cause of bronchiectasis. Accurate PCD diagnosis comprises clinical assessment, analysis of cilia and the identification of biallelic variants in one of 50 known PCD-related genes, including *HYDIN*. *HYDIN*-related PCD is underdiagnosed due to the presence of a pseudogene, *HYDIN2*, with 98% sequence homology to *HYDIN*. This presents a significant challenge for Short-Read Next Generation Sequencing (SR-NGS) and analysis, and many diagnostic PCD gene panels do not include *HYDIN*. We have used a combined approach of SR-NGS with bioinformatic masking of *HYDIN2*, and state-of-the-art long-read Nanopore sequencing (LR_NGS), together with analysis of respiratory cilia including transmission electron microscopy and immunofluorescence to address the underdiagnosis of *HYDIN* as a cause of PCD. Bioinformatic masking of *HYDIN2* after SR-NGS facilitated the detection of biallelic *HYDIN* variants in 15 of 437 families, but compromised the detection of copy number variants. Supplementing testing with LR-NGS detected *HYDIN* deletions in 2 families, where SR-NGS had detected a single heterozygous *HYDIN* variant. LR-NGS was also able to confirm true homozygosity in 2 families when parental testing was not possible. Utilising a combined genomic diagnostic approach, biallelic *HYDIN* variants were detected in 17 families from 242 genetically confirmed PCD cases, comprising 7% of our PCD cohort. This represents the largest reported *HYDIN* cohort to date and highlights previous underdiagnosis of *HYDIN*-associated PCD. Moreover this provides further evidence for the utility of LR-NGS in diagnostic testing, particularly for regions of high genomic complexity.

## Introduction

Primary ciliary dyskinesia (PCD) comprises a group of genetically heterogenous disorders of the motile (9 + 2) cilia that contributes to a growing number of ciliopathies [1]. There is increasing awareness that PCD is underdiagnosed as a cause of bronchiectasis and therefore its accurate and comprehensive diagnosis is essential [2, 3]. Individuals with PCD present with a spectrum of clinical findings, including neonatal respiratory distress, chronic upper and lower respiratory tract disease, sinus and ear infections, laterality defects, and infertility [4–8]. Phenotypic heterogeneity, influenced by the underlying genetic cause, is observed among individuals with PCD [4]. Recent work has estimated the global prevalence of PCD is approximately 1:7500 individuals, although the prevalence has been shown to vary greatly among different populations [9].

PCD is almost exclusively an autosomal recessive condition, however, rare X-linked forms and an autosomal dominant form have been reported [10]. Currently, pathogenic variants in at least 50 genes encoding various components of the cilia as well as trafficking proteins are known to cause the condition [11–13]. Pathogenic variants in these genes usually cause associated defects in the axonemal ultrastructure of the motile cilia, or, more rarely, reduced cilia numbers [13]. Disruption in these cilia components typically causes defects in ciliary motility and waveform that characterise the motile ciliary dysfunction observed in individuals with PCD. Of note, some genes, such as *DNAH11* [14], are known to be associated with normal axonemal ultrastructure, often due to subtle defects that are missed by clinical transmission electron microscopy (TEM).

In view of the highly variable clinical phenotypes and genetically heterogenous ciliary disruptions observed in PCD, diagnosis requires a specialist multidisciplinary approach. Current guidelines for PCD diagnosis recommend integration of nasal nitric oxide concentration measurement, cilia structure-function assessment by high-speed video microscopy analysis (HSVMA), immunofluorescence (IF), TEM and genotyping [15, 16].

Currently, a genetic diagnosis is made in up to 75% of clinically confirmed cases, following detailed clinical and cilia studies [12]. Although PCD is genetically heterogeneous, it is well documented that pathogenic variants in some genes are observed more frequently than in others, although this distribution differs between populations [9, 17]. For example, biallelic variants in *DNAH11* and *DNAH5* are the most common causes of PCD in Europe, accounting for ~30% of genetically confirmed cases [9]. Mutations in *CCDC39*, *CCDC40* and *DNAI1* are also recognised as common causes of PCD whilst mutations in the remaining PCD-associated genes are identified more rarely [9, 12, 18].

The *HYDIN* gene was first described in mice as a recessive cause of hydrocephalus, with its expression localised to the motile cilia [19]. *HYDIN* encodes the Hydrocephalus-inducing protein homologue and its expression within the motile cilia was further localised to a single projection from the C2 microtubule of the central pair apparatus, called C2b [20, 21]. The motile cilia in homozygous *HYDIN* mutant mice are reported to be unable to bend fully and thus have a significantly reduced cilia beat frequency, which leads to impaired fluid flow in the brain and the development of hydrocephalus [21, 22]. In addition, similar ciliary beat defects were observed in mouse tracheal cilia, suggestive of a potential role for *HYDIN* as a cause of PCD [21, 22].

*HYDIN* (OMIM 610812), located on chromosome 16q22.2 in humans [19], encodes the HYDIN axonemal central pair apparatus protein and biallelic *HYDIN* variants cause PCD in humans [22–25]. Unlike most other PCD genes, recessive *HYDIN* variants do not cause laterality defects, but do otherwise present with typical clinical findings associated with PCD. When observed by HSVMA, cilia on nasal epithelial cells from patients with *HYDIN* variants show abnormal axonemal bending, as observed in mouse models, which results in a twisting/rotating appearance similar to the beat pattern of 9 + 0 nodal cilia, which lack a central pair complex [22]. Although loss of the C2b projections also occurs in these patients, the small size of this projection from the central pair complex means that it is rarely possible to visualise this absence on TEM, although this is possible to demonstrate using 3D electron microscopy tomography [22]. In clinical screening, IF must be used instead [22]. However, there is a lack of commercially available antibodies for the *HYDIN* protein within C2b. In addition to C2b, the central pair complex is known to have 6 further projections, including C1b, which anchors the C2b projection to the C1 microtubule [26]. It has been shown that a component of the C1b projection, Sperm Flagellar 2 encoded by the *SPEF2* gene, associates directly with *HYDIN*, and that loss of *HYDIN* causes concurrent loss of *SPEF2*. Consequently, IF using antibodies for *SPEF2* has been found to be informative for patients with *HYDIN* variants, where loss of *SPEF2* staining is apparent [27].

Humans carry a paralogous copy of *HYDIN* named *HYDIN2* (OMIM 610813, HYDIN axonemal central pair apparatus protein 2) located on chromosome 1q21.2 [28]. This 360 kb duplication includes exons 6–84 of the *HYDIN* gene, with only the first 5 and final 2 exons being unique. The level of homology between the duplicated exons of *HYDIN* and *HYDIN2* exceeds 98% across the entire region [28]. The presence of *HYDIN2* introduces problems with genetic analysis, since the shared homologous regions make it difficult to design PCR primers that uniquely amplify target regions, to create probes to capture regions of interest for short-read sequencing, or to uniquely map short reads after sequencing. Due to this genomic complexity, *HYDIN* is not included in many PCD diagnostic gene panels [27, 29]. These challenges along with the lack of laterality defects, absence of clearcut diagnostic cilia structural defects, and relative preservation of cilia motility in affected individuals all contribute to the underdiagnosis of *HYDIN*-related PCD.

Short-read sequencing technology is generally limited in its ability to identify structural variants, to sequence repetitive regions, to phase alleles, and to distinguish highly homologous genomic regions [30]. We hypothesised that the relatively low number of pathogenic variants in *HYDIN* reported in individuals with PCD may be due to technical and analytical difficulties in analysing *HYDIN* because of its similarity with *HYDIN2;* and that long-read sequencing (LRS) could be used to identify missing disease-causing variants in these cases [31, 32]. In this paper, we use a combination of short-read and long-read sequencing to identify likely disease-causing variants in *HYDIN* in 17 families who lacked a precise genetic diagnosis, comprising 7% of our PCD diagnostic cohort.

## Subjects and methods

### Individuals and samples

The study cohort comprised individuals with a clinical suspicion of PCD, from 437 families who had been referred for molecular genetic diagnostic testing and, in most cases, analysis of their respiratory cilia. All patients were recruited at the Royal Brompton Hospital and provided written informed consent for genetic testing and the use of their data for research. Ethics approval for genetic studies was obtained from the NHS Health Research Authority, IRAS project ID: 103488 and London-Bloomsbury Research Ethics Committee (REC) reference: 08/H0713/82. DNA was extracted from peripheral EDTA blood or saliva from patients using the QIAGEN EZ1 Advanced XL or QIAGEN QIAsymphony instrument, following the manufacturer’s protocol.

### Cilia diagnostics

Following a detailed assessment of clinical features and presentation, nasal nitric oxide levels were measured in all individuals >5 years old by chemiluminescence (Logan 2500, Logan Sinclair, Kent, UK) or for patients after 2020 electrochemically (Niox Vero, Circassia). Readings from each nostril were recorded during velum closure manoeuvres (breath holding or breathing against a resistance) and the average value recorded in ppb. Where possible results were converted to nl/min for reporting, or if conversion was not possible (for tidal nasal nitric oxide measurements) results were ported in ppb. All patients underwent a nasal brushing for PCD diagnosis and ALI cell culture was set up as described in Supplementary Material.

High-speed video microscopy was performed on fresh epithelial strips in a chamber slide at 37 °C using a 100× oil immersion objective and Leica upright microscope (DM-LB) with high-speed video camera (Troubleshooter TS-5 Fastec imaging) as described in supplementary material. Ten strips of ciliated epithelium were recorded, including top and side views, and assessed by a diagnostic scientist for beat pattern and frequency as previously described [33]. Samples were subsequently fixed in cacodylate buffered 2.5% glutaraldehyde for transmission electron microscopy (TEM). Electron microscopy was conducted as previously described and summarised in Supplementary Material. 300 ciliary cross sections were counted per section and results reported according to the BEAT-PCD TEM consensus guideline [34]. In cases where variants in *HYDIN* were suspected as a cause, advanced TEM techniques were employed to visualise the C2b projection. These included electron tomography [22] or image averaging via an inhouse developed program (PCD detect) [34].

Samples taken after 2020 were air dried onto slides and stained for SPEF2 by immunofluorescence (supplementary material). Ten cells were assessed per sample, and the co-localisation of SPEF2 protein with acetylated tubulin of the ciliary axoneme was recorded as present or absent.

### Genetic diagnosis

#### Targeted short-read next generation sequencing

Targeted short-read NGS (SR-NGS) was performed on a custom 182-gene panel, using Agilent SureSelect QXT library preparation and sequencing on a NextSeq550 platform (Illumina, San Diego, USA). Library preparation used a paired-end protocol, resulting in fragment lengths between 150–300 bp. Negative controls were added to each library prep to ensure minimal contamination occurred. Sequence data from targeted SR-NGS was analysed using an automated in-house bioinformatics pipeline (details provided in supplementary material). For individuals from all 437 families, first line SR-NGS analysis was targeted to 47 genes associated with PCD, including all coding exons of the *HYDIN* gene. The *HYDIN2* region (chr1:146472566-146914294, GRCh38 reference) was programmatically masked using bedtools v2.27.0 maskfasta feature, so that all four alleles of *HYDIN* and *HYDIN2* were aligned to *HYDIN*. Variants were filtered and classified according to in-house decision trees, which included multiple parameters, such as allele frequency in the gnomAD database (http://exac.broadinstitute.org; www.gnomad.org), presence in HGMD, presence in our in-house variant database, ClinVar (https://www.ncbi.nlm.nih.gov/clinvar/) and PubMed (https://www.ncbi.nlm.nih.gov/pubmed/). Missense variants were assessed for their effect on protein structure and function using SIFT, Polyphen2, LRT, Grantham score, MutationTaster, MutationAssessor and FATHMM. NNSplice, MaxEntScan, SpliceSiteFinder-like and SpliceAI [35] were used to assess the impact of variants potentially affecting splicing. Copy number variants (CNVs) were called from SR-NGS data using an ISO15189-accredited and validated in-house method based on read-depth analyses of all targeted exons (further bioinformatic details are provided in supplementary material).

Manual review and final variant classification was performed by a Clinical Scientist according to the ACMG/AMP guidelines [36] with subsequent modifications [37, 38]. The results of all clinical, cilia and genetic tests were discussed at a monthly multidisciplinary meeting.

#### Targeted long-read sequencing

Long-read sequencing (LRS) was performed on the Oxford Nanopore Technologies (ONT) platform for individuals from 4 families where SR-NGS alone was unable to confirm a genetic diagnosis. Libraries for sequencing were prepared using the Oxford Nanopore ligation kit (SQK-LSK110; further details provided in Supplementary Materials and Methods). Libraries for targeted LRS (T-LRS) were loaded onto a R9.4.1 flow cell on a Nanopore GridION running MinKNOW version 21.10.8. Adaptive sampling was performed using ReadFish to target *HYDIN* (chr16:70300000–71700000) and *HYDIN2* (chr1:146000000–147000000), as well as two control regions (*COL1A1*, chr17:50000000–50250000 and *FMR1*, chrX:147800000–148000000) using GRCh38 as the reference (https://pubmed.ncbi.nlm.nih.gov/33257864/).

Raw sequencing data was base called with Guppy 5.0.12 (ONT) using the super accurate (SUP) model with 5mC modification detection. FASTQs were generated from unaligned bam files using Samtools Fastq [39] and aligned to GRCh38 using minimap2 [40]. Depth of coverage for *HYDIN* and *HYDIN2* was calculated using Samtools depth. Single nucleotide and indel variants were called using Clair3 [41] then phased using LongPhase using variant calls from Clair3 [42]. Single nucleotide (SNVs) and indel variants were annotated using VEP [43] including CADD and SpliceAI scores as well as allele frequency from gnomad version 3 [44, 45]. Structural variants (SVs) were called using Sniffles2, CuteSV, and SVIM [46–48]. Variants in *HYDIN* with allele frequencies in gnomad less than 1% or that had never been observed before as well as all SVs were prioritised for analysis. Compelling variants were visualised using IGV [49].

#### Targeted variant testing and primer design

Targeted testing for SNVs and indels identified by both SR-NGS and LRS was performed using bidirectional Sanger sequencing (details provided in supplementary material). Any potential CNVs identified by SR-NGS and LRS were confirmed by digital droplet PCR (ddPCR) (BioRad, CA, USA) (supplementary material).

## Results

### Characteristics of the cohort

A clinical diagnosis of PCD was based on nasal nitric oxide measurement and a history or presence of clinical features such as neonatal respiratory distress in a term infant, chronic productive wet cough, bronchiectasis, rhinorrhoea, and serous otitis media. Some individuals had cilia analysis on nasal brushing samples (Table 1). As previously described for PCD caused by pathogenic variants in *HYDIN*, none of the individuals in the families found to have at least one pathogenic variant in *HYDIN* presented with laterality defects.

**Table 1:** Phenotypic information and results of clinical diagnostic testing in affected individuals from the 17 families reported here.

Family	Individual	Age at presentation	Clinical findings	Clinical laboratory testing
Family 1	II:4	22 years	Bronchiectasis, recurrent LRTI, chronic productive cough	- NNO: 152.5 nl/min - HSVM: Stiff with mixed beat pattern
	III:3	Birth	Respiratory distress at birth, LRTI’s, bronchiolitis, chronic wet cough, glue ear, rhinitis	- NNO: 26ppb (tidal) - HSVM: Stiff and uncoordinated with circling observed - EM: Normal ultrastructure with ‘smudging’ of central pairs
	III:4	Birth	Respiratory distress at birth, LRTI’s, rhinitis	- NNO: 66ppb (tidal) - HSVM: Stiff and uncoordinated with circling observed
	III:5	Birth	Respiratory distress at birth, LRTI’s, ear infections	- HSVM: Stiff and uncoordinated - EM: Normal ultrastructure with ‘smudging’ of central pairs
	III:6	Birth	Chronic productive cough, LRTI’s	- HSVM: Stiff and uncoordinated
Family 2	II:5	Unknown	Bronchiectasis and LRTI	- NNO: 29 nl/min
	II:6	Unknown		- NNO: 29 nl/min - HSVM: Predominantly stiff, reduced beat amplitude and weak residual movement
Family 3	II:1	Unknown	Wet cough, recurrent otitis media. Hearing problems	- HSVM: Stiff and reduced beat amplitude some subtle rotation - EM: Absence of some central pairs
	II:2	Unknown	Chronic productive cough, glue ear	- NNO: 80 nl/min - HSVM: Stiff and reduced beat amplitude some subtle rotation
Family 4	II:1	3 years	Chronic bronchiectasis and rhinosinusitis	- NNO: 50 nl/min - HSVM: Dyskinetic with a circling beat pattern - EM: Disarrangement
	II:2	5 years	Bronchiectasis, rhinorrhoea	- Not undertaken
Family 5	II:1	Birth	Chronic wet cough, Rhinitis	- NNO:25 nl/min - HSVM: Dyskinetic with a circling beat pattern - EM: Absence of some central pairs - IF: Loss of *SPEF2* staining
	II:2	Birth	Neonatal pneumonia, chronic wet cough, LRTI’s, ear infections, rhinorrhoea	- NNO:15/min - HSVM: Dyskinetic with a circling beat pattern - EM: Absence of some central pairs - IF: Loss of *SPEF2* staining - Tomography: Absence of C2b protein
Family 6	II:1	Unknown	Bronchiectasis, Type II respiratory failure	- Not undertaken
Family 7	II:1	12	Bronchiectasis	- NNO: 16 nl/min - HSVM: Stuff with circling patches - EM: Normal ultrastructure - IF: Loss of *SPEF2* staining
	II:2	5	Bronchiectasis, rhinitis	- Not undertaken
Family 8	II:1	Birth	Respiratory distress, ear infections, rhinitis, glue ear and impaired hearing	- NNO: 11 nl/min - HSVM: Stiff and uncoordinated beat with circling observed - EM: Normal ultrastructure - Tomography: Absence of C2b protein
Family 9	II:1	Birth	Bronchiectasis	- HSVM: Stiff and uncoordinated beat with circling observed - EM: Absence of some central pairs
Family 10	II:1	Birth	Bronchiolitis, glue ear, chronic wet cough	- NNO: 38 nl/min - HSVM: Dyskinetic with circling observed - EM: Normal ultrastructure - IF: Loss of *SPEF2* staining
Family 11	II:1	Birth	Bronchiectasis, rhinitis, glue ear	- NNO: 11 nl/min - HSVM: Stiff and dyskinetic - EM: Normal ultrastructure
Family 12	II:1	Birth	Bronchiectasis, chronic sinusitis and poor hearing	- NNO: 15 nl/min - HSVM: Stiff and dyskinetic with a circling beat pattern - EM: Absence of some central pairs - IF: Loss of SPEF2 staining
Family 13	II:1	27	Mild bronchiectasis, chronic rhinosinusitis, polyps, glue ear, fertility difficulties	- NNO: 6 nl/min - HSVM: Uncoordinated, stiff - EM: Normal ultrastructure
	II:2	29	Bronchiectasis, ear infections, glue ear, fertility difficulties	- NNO: 42 nl/min - HSVM: Uncoordinated, stiff, weak residual movement, occasional rotation - EM: Normal Ultrastructure - IF: *SPEF2* present
Family 14	II:1	Unkown	Rhinorrhoea, chronic productive cough	- NNO: 4 nl/min - HSVM: Stiff with circling observed - EM: Normal ultrastructure - IF: Loss of *SPEF2* staining
Family 15	II:1	3 years	Chronic productive cough	- NNO: 170 nl/min - HSVM: Stiff with circling observed - EM: Absence of some central pairs
Family 16	II:1	13 years	Bronchiectasis	- NNO: 14 - HSVM: Circling observed - EM: Normal ultrastructure
Family 17	II:1	Unknown	Chronic wet cough, bilateral hearing aids	- NNO: 31ppb (tidal) - HSVM: Stiff with circling observed - EM: Normal ultrastructure

*NNO* Nasal Nitric Oxide, *HSVM* High Speed Videomicroscopy, *EM* Electron Microscopy, *LRTI* Lower Respiratory Tract Infections.

### Short read NGS to identify pathogenic variants in *HYDIN*

**Fig. 1:**
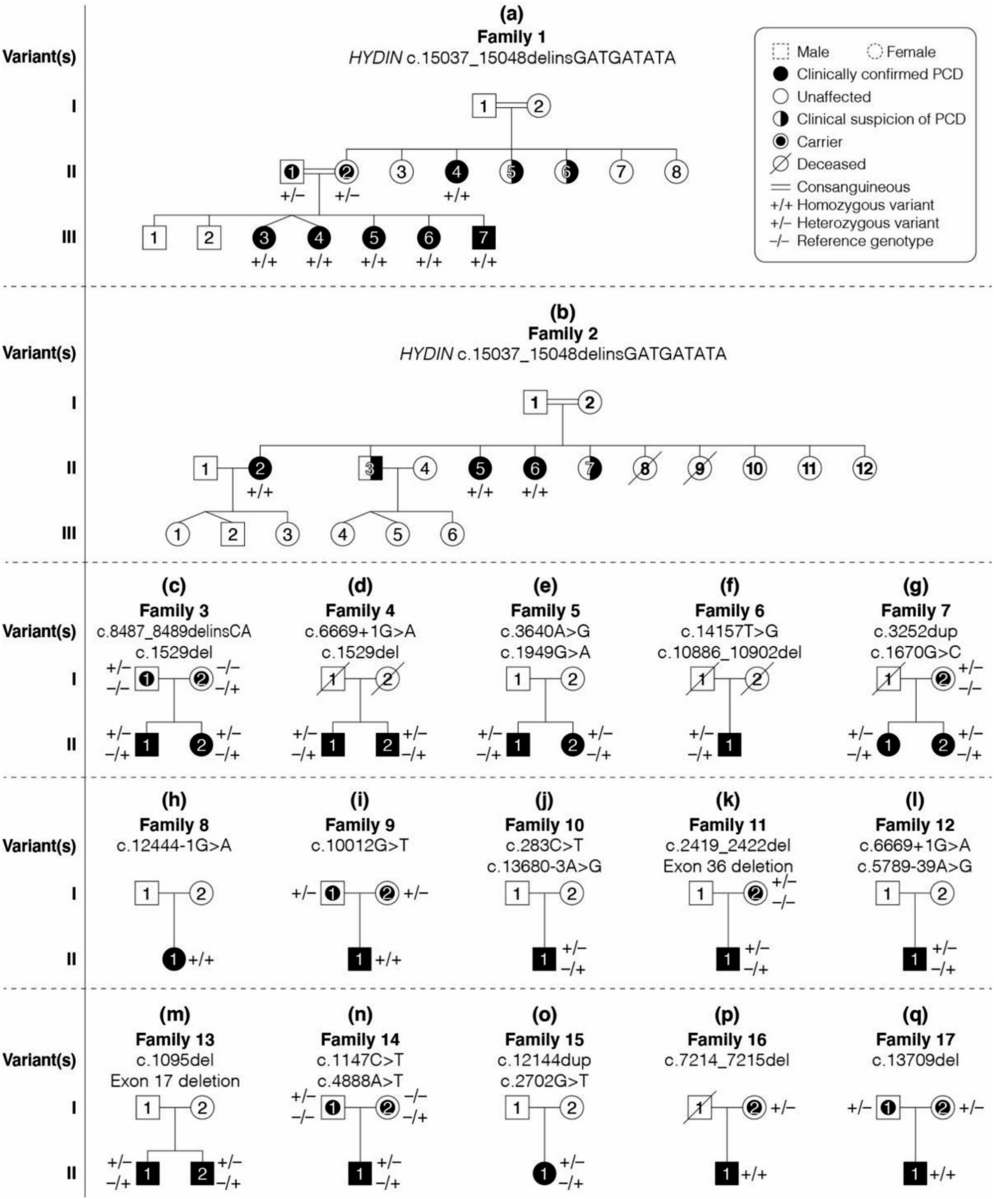
Pedigrees of the 17 families in whom *HYDIN* variants were found to be causative of PCD. The variants identified in each of the families are listed with each pedigree (**a**–**q**). Circle: Female; Square: Male; Filled symbols: confirmed clinical diagnosis of PCD; Half-filled symbols: clinical suspicion of PCD; Dot symbol: Carrier; Slash through: Deceased; +/+: homozygous variant; +/−: heterozygous variant; −/−: reference genotype.

A custom panel was used to evaluate individuals from all 437 families, and this identified potentially disease-causing variants in one of the 47 PCD-associated genes in 242 families. Candidate pathogenic variants in *HYDIN* were found in 17 unrelated families comprising 29 affected individuals (Fig. 1, Table 2). Sixteen individuals from 11 of these families were compound heterozygous for the identified variants in *HYDIN*, while 13 individuals in six families where consanguinity was known or suspected, were homozygous for a single variant. Where possible, parental testing was performed to phase the identified variants (Fig. 1, Table 2). The majority of *HYDIN* variants identified in these families were truncating or splice-site variants that were classified as pathogenic or likely pathogenic based on ACMG criteria [36]. Three missense and one potential splice variant were classified as variants of uncertain significance (VUS).

**Table 2:** Summary of variants identified as the cause of PCD in 17 families, together with their ACMG classification.

Family	Variant	Variant (cDNA)	Variant (amino acid)	ACMG classification	Individuals found in
Family 1	1, 2	c.15037_15048delinsGATGATATA	p.(Tyr5013_Pro5016 delinsAspAsplle)	LP	II:1, II:2, II:4, III:3, III:4, III:5, III:6, III:7
Family 2	1, 2	c.15037_15048delinsGATGATATA	p.(Tyr5013_Pro5016 delinsAspAsplle)	LP	II:2, II:5: II:6
Family 3	1	c.8487_8489delinsCA	p.(Pro2830Hisfs*23)	LP	I:1, II:1, II:1
	2	c.1529del	p.(Phe510Serfs*43)	P	I:2, II:1, II:1
Family 4	1	c.1529del	p.(Phe510Serfs*43)	P	II:1, II:2
	2	c.6669+1G>A	p.?	LP	II:1, II:2
Family 5	1	c.1949G>A	p.(Arg650His)	VUS	II:1, II:2
	2	c.3640A>G	p.(lle1214Val)	VUS	II:1, II:2
Family 6	1	c.10886_10902del	p.(Asp3629ValfsTer9)	LP	II:1
	2	c.14157T>G	p.(Tyr4719Ter)	LP	II:1
Family 7	1	c.3252dup	p.(Val1085ArgfsTer15)	P	I:2, II:1, II:2
	2	c.1670G>C	p.(Arg557Thr)	LP	II:1, II:2
Family 8	1,2	c.12444-1G>A	–	LP	II:1
Family 9	1,2	c.10012G>T	p.(Glu338Ter)	P	I:1, I:2, II:1
Family 10	1	c.283C>T	p.(Gln95Ter)	LP	II:1
	2	c.13680-3A>G	p.?	VUS	II:1
Family 11	1	c.2419_2422del	p.(Val807llefsTer13)	LP	I:2, II:1
	2	c.5620-311_5788+1198del	p.?	LP	II:1
Family 12	1	c.6669+1G>A	p.?	LP	II:1
	2	c.5789-39A>G	p.?	VUS	II:1
Family 13	1	c.1095del	p.(Phe365LeufsTer64)	LP	II:1, II:2
	2	c.2376+752_2529+9004del	p.?	LP	II:1, II:2
Family 14	1	c.1147C>T	p.(Arg383Ter)	P	I:1, II:1
	2	c.4888A>T	p.(Lys1630Ter)	P	I:2, II:1
Family 15	1	c.12144dup	p.(Thr4049HisfsTer9)	LP	II:1
	2	c.2702G>T	p.(Gly901Val)	VUS	II:1
Family 16	1,2	c.7214_7215del	p.(Ser2405CysfsTer2)	LP	I:2, II:1
Family 17	1,2	c.13709del	p.(Pro4570LeufsTer22)	P	I:1, I:2, II:1

*LP* LP, *P* P, *VUS* variant of uncertain significance.

**Fig. 2:**
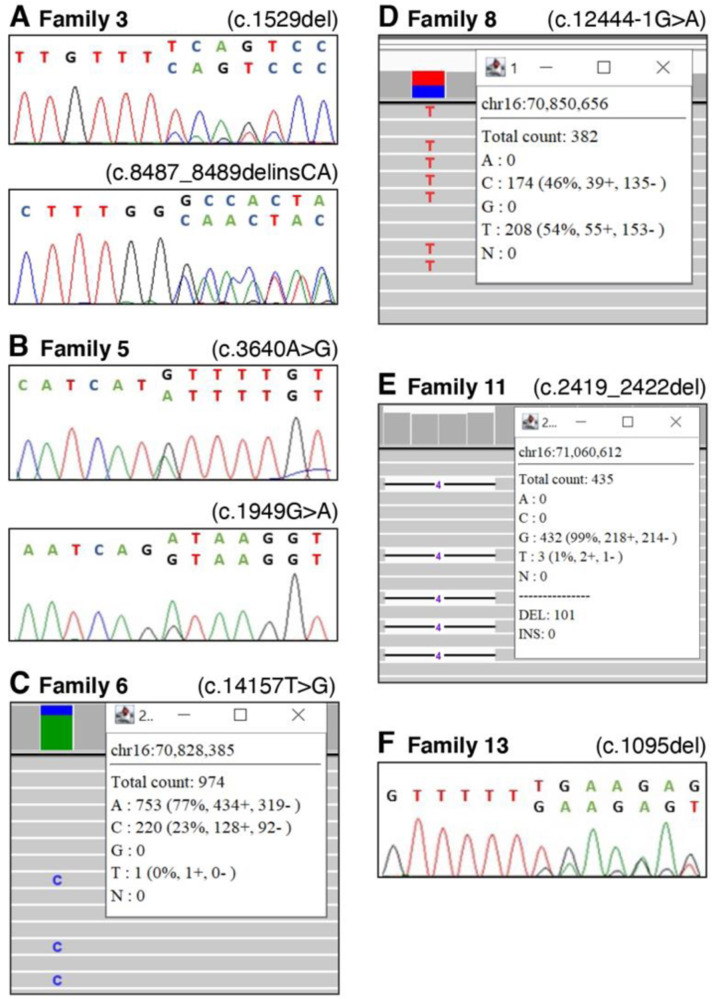
Sequence analysis of variants of note identified in this *HYDIN* PCD cohort. **A** Sanger sequencing of variant c.1529del in family 3 showing a heterozygous result where primer design specifically for *HYDIN* was not possible, resulting in low allelic ratio of the variant. Sanger sequencing of variant c.8487_8489delinsCA in a heterozygous individual from family 3 where primer design specifically for *HYDIN* was possible and the allelic ratio is 50%. **B** Sanger sequencing of variants c.3640A>G and c.1949G>A in family 5, both showing skewed allelic ratios in heterozygous individuals. **C** SR-NGS example for variant c.14157T>G in a heterozygous individual from family 6 showing skewed allelic ratio due to *HYDIN2* masking. **D** SR-NGS example for variant c.12444-1G>A in a homozygous individual from family 8, showing skewed allelic ratio due to HYDIN2 masking. **E** SR-NGS of c.2419_2422del detected in a heterozygous individual in family 11, showing skewed allelic ratio due to *HYDIN2* masking. **F** Sanger sequencing of variant c.1095del in a heterozygous individual from family 13, showing skewed allelic ratios.

Consistent with the high sequence homology between *HYDIN* and *HYDIN2*, the majority of variants detected in our cohort were in exons present in both genes and thus demonstrated skewed allele balance by SR-NGS. Of the 24 different *HYDIN* variants reported here, only c.283C>T p.(Gln95Ter) in family 10 and c.15037_15048delinsGATGATAT p.(Tyr5013_Pro5016delinsAspAsplle) in families 1 and 2 were within sequences unique to *HYDIN*, being located in exons 4 and 86 respectively. These variants had allelic ratios of approximately 50%, as assessed by SR-NGS and Sanger sequencing. Due to the masking of *HYDIN2* prior to read alignment, the majority of the remaining variants demonstrated skewed allelic balances in SR-NGS and Sanger sequencing (Fig. 2A variant c.1529del, B, F), with a heterozygous variant present in 25% of reads rather than 50% (Fig. 2C) and a homozygous variant being present in 50% of reads rather than 100% (Fig. 2D). For a small number of variants it was possible to design primers utilising known sequence differences between *HYDIN* and *HYDIN2*, to produce *HYDIN-*specific sequencing and a normal 50% level of heterozygous allelic balance (Fig. 2A, variant c.8487_8489delinsCA).

Of the *HYDIN* variants identified, only three have previously been reported in individuals with PCD (c.6669+1G>A [24], c.10012G>T [17] and c.1147C>T [50]) (Table 2). Of the previously reported variants, only the c.6669+1G>A variant is present in population databases (15/398,136 alleles in gnomAD, v2 and v3) and was detected in two families in our cohort, indicating it is likely a relatively common pathogenic *HYDIN* variant. Even within our large cohort of *HYDIN* PCD families, only two variants, the c.6669+1G>A variant and the previously unreported c.1529del variant, were identified in more than one family (Table 2), suggesting that most disease-causing *HYDIN* variants are likely to be private. In families 1 and 2, who were both referred to our laboratory from Northern Ireland, the same homozygous in-frame indel variant, c.15037_15048delinsGATGATATA, was found. Although these families are not known to be closely related, they are all part of the same Irish traveller community, suggesting it may represent a founder variant in this population.

**Fig. 3:**
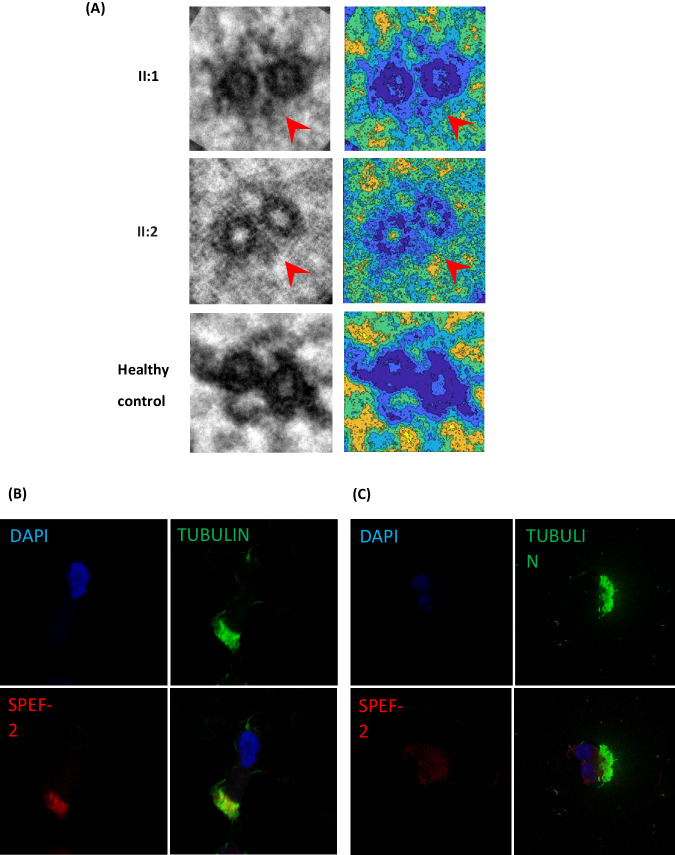
Nasal Cilia brushing diagnostics for siblings in family 5. **A** Averaged electron microscopy image and corresponding coloured contour map showing absence of c2b protein in these siblings, II-1, II-2 and a healthy control. **B** Immunofluorescence control sample with DAPI staining of the nucleus, tubulin staining of the cilia and SPEF2 for the c2b protein. **C** Immunofluorescence showing loss of SPEF2 staining in II:1.

As previously observed in *HYDIN*, as well as in other genes associated with PCD, the majority of variants reported in these families were loss-of-function variants (~85%), with missense variants being observed only in families 5, 7, and 15. Of note, in family 5, no truncating variants were detected, and both affected siblings were found to be compound heterozygous for two missense variants in *HYDIN*, c.1949G>A and c.3640A>G (Fig. 2B). Nasal brushing TEM and HSVM results were consistent with a *HYDIN* phenotype, as a complete loss of *SPEF2* staining was observed by IF and absence of the C2b projection of the central pair microtubular complex was observed by 3D tomography in the affected siblings (Table 1, Fig. 3A–C). Moreover, the typical rotational beat pattern of HYDIN-deficient cilia was observed (Supplementary Fig. 1). Although these variants were both classified as VUS, in the absence of other *HYDIN* variants in these siblings, we consider these variants likely to be the cause of their PCD. Separately, the proband in family 12 was found to be compound heterozygous for two variants, each affecting splicing: the previously reported c.6669+1G>A variant and a deeper intronic variant, c.5789-39A>G. Splice prediction tools, including SpliceAI, suggested that the c.5789-39A>G variant would create a new splice acceptor site in intron 36, which would lead to the inclusion of an additional 38 nucleotides in the transcript, introducing a frameshift and likely inducing nonsense-mediated decay. The inclusion of additional intronic sequence to the beginning of exon 37 was confirmed in cDNA from the patient.

### Clarifying previously identified variants using long-read sequencing

**Fig. 4:**
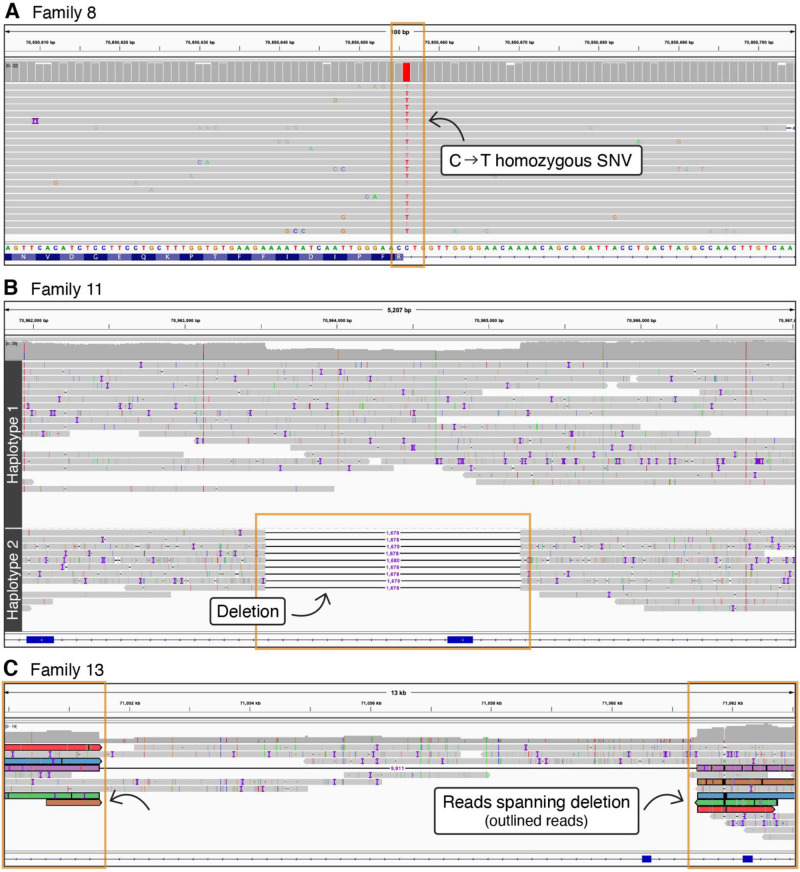
LR-NGS in this *HYDIN* PCD cohort. **A** Confirmation of homozygosity by LR-NGS for the c.12444-1G>A variant in family 8. **B** *HYDIN* coding exon 36 deletion (chr16:70963530-70965207) detected by LR-NGS in family 11. **C** *HYDIN* coding exon 17 deletion (chr16:71051501-71061418) detected by LR-NGS in family 13.

In the absence of parental samples to confirm phasing in Family 8, targeted LRS was used to confirm homozygosity for the c.12444-1G>A variant. Figure 4A shows the presence of the homozygous G>A change in *HYDIN* at c.12444-1 (represented as C>T). This is consistent with the 46%:54% C:T allelic ratio observed in Fig. 2D, with approximately half of the reads contributed by *HYDIN2 (*due to the masked alignment), which has a normal C at this position. SR-NGS of proband II:1 in family 16 identified a homozygous frameshift variant, c.7214_7215del p.(Ser2405CysfsTer2), and a heterozygous frameshift variant, c.7956dup p.(Glu2653ArgfsTer26), both of which occurred in a region of high homology between the two genes. Homozygosity for the c.7214_7215del was consistent with the reported consanguinity in the family, and we considered it highly unlikely that only one parent had both the c.7214_7215del and c.7956dup variants *in cis*. This suggested that one of the two variants may be in *HYDIN2*. The proband’s mother was found to be heterozygous for the c.7214_7215del variant and not the c.7956dup variant by Sanger sequencing, however, paternal testing was not possible. Using targeted LRS, we confirmed that the proband was indeed homozygous for the c.7214_7215del variant in *HYDIN* and that the c.7956dup was present in *HYDIN2*.

### Long-read sequencing to identify missing disease-causing variants

First-line SR-NGS testing in the probands of families 11 (II:1) and 13 (II:1 and II:2) detected a single likely pathogenic variant in *HYDIN*: c.2419_2422del p.(Val807llefsTer13) in family 11 and c.1095del p.(Phe365LeufsTer64) in the two affected siblings in family 13 (Fig. 2E, F, Table 2). Due to the high clinical suspicion for PCD, we questioned whether these families harboured variants that were difficult to detect by prior testing approaches. Targeted LRS was therefore used to identify second hits, both CNVs, in both families (Fig. 4B, C). This approach resulted in approximately 2–5× enrichment of the target regions, with coverage of both *HYDIN* and the pseudogene *HYDIN2,* and allowed us to evaluate the region for candidate pathogenic variants in *HYDIN* (Supplementary Table 1). In family 11 a heterozygous likely pathogenic 1678-bp deletion that included the 36^th^ coding exon of *HYDIN* (chr16:70963530-70965207) was identified that would result in a frameshift and subsequent premature termination codon early in exon 37. In family 13 a heterozygous 9900-bp deletion that included the 17^th^ coding exon of *HYDIN* (chr16:71051501-71061418) was found. While the exon 17 deletion is in-frame it would remove 50 highly conserved amino acids, and therefore it is likely to alter protein function. Both deletions were confirmed by ddPCR of the relevant exons. Exons 17 and 36 both lie within a region of high homology between *HYDIN* and *HYDIN2* and were not detected by initial SR-NGS CNV analysis, due to the skewed allelic balance resulting from the masking of *HYDIN2*.

## Discussion

In a cohort of 437 unrelated families referred for genetic testing with a clinical diagnosis or strong clinical suspicion of PCD, we were able to diagnose 29 affected individuals from 17 unrelated families as carrying potentially pathogenic variants in *HYDIN*. In four of the 17 families, a complete genetic diagnosis was only possible using LRS. This study comprises the largest *HYDIN* cohort reported to date and confirms that comprehensive genetic testing using different techniques can be used to identify variants in challenging regions of the genome. *HYDIN* is revealed as a relatively common cause of PCD in our cohort, representing ~7% of our genetically diagnosed cases. This is in line with a recent report of *HYDIN* being shown to be causative in 8.7% of families with PCD in Quebec, Canada, although 5 of 8 families in that study shared the same founder variant [29].

While advances in molecular genetic testing have revolutionised the approach to diagnosis of individuals with suspected genetic disorders, several notable challenges remain. One example includes the difficulty associated with analysing repetitive or highly homologous regions of the genome, such as those observed in *HYDIN* and *HYDIN2*. Initially, we addressed this problem computationally by masking *HYDIN2* during sequence alignment to ensure that reads from *HYDIN* and *HYDIN2* would be mapped to *HYDIN*. This approach ensures that no *HYDIN* sequence is incorrectly mapped to *HYDIN2*, and therefore eliminates the possibility of true *HYDIN* variants being excluded from analysis. Although this approach overcomes mapping inconsistencies, it results in skewed variant allelic balances, since there are four potential copies of the sequence, two from *HYDIN* and two from *HYDIN2*, at positions of homology. However, the identification of variants using skewed allele balance allows for subsequent analysis of candidate variants by targeted approaches such as PCR, SR-NGS and Sanger sequencing, with heterozygous variants having an allelic balance of 0.25 and homozygous variants having an allelic balance of 0.5.

Masking of *HYDIN2* overcame some of the difficulties in the detection of single nucleotide variants and small indels by SR-NGS, as demonstrated by our ability to identify disease-causing variants in 14/17 families in this study. However, as highlighted by families 11 and 13, this method has limitations with detecting deletions spanning exons, since masking interferes with NGS CNV-calling algorithms. After masking of *HYDIN2*, we expected to observe a ~0.25 allelic ratio when a heterozygous deletion was present in either gene. This is likely not sufficient for detection by standard short-read CNV callers, a fundamental limitation of short-read sequencing. It is important to note that generally SR-NGS alone is unable to confirm exactly which gene is affected when a variant is identified in the highly homologous regions of *HYDIN* or *HYDIN2*, although effective phenotyping does increase the confidence that variants in these cases lie within the *HYDIN* gene.

We hypothesised that long-read sequencing could be used to identify missing variants or refine the classification of candidate variants in cases refractory to our standard approaches. This is because the longer reads generated by this technology are more likely to be accurately mapped to low-complexity or repetitive regions, such as the regions in which *HYDIN* and *HYDIN2* are found. We also hypothesised that LR-NGS would identify variants we were not able to identify with SR-NGS, such as intronic variants and structural variants. Using a targeted approach, we were able to identify a second disease-causing variant in 2 families where SR-NGS identified only a single heterozygous pathogenic variant. Specifically, LR-NGS in families 11 and 13, identified deletions of coding exons 36 and 17 respectively, which were missed by SR-NGS because of masking of *HYDIN2* and difficulty identifying CNVs with allele frequencies of 0.25. In a third individual, where parental testing was not possible, we used LR-NGS to confirm that the identified pathogenic variant was indeed homozygous (family 8). It is likely that LR-NGS would have been able to detect all the variants reported in the other families and thus would offer additional benefits over SR-NGS, such as confirming phasing of variants without the need for parental samples.

We have presented two cases where LR-NGS was able to supplement SR-NGS and detect a missed second variant in *HYDIN*. For such individuals, with a phenotype highly in keeping with *HYDIN*, LR-NGS may be indicated following an in normal or incomplete SR-NGS result. Based on the cohort included here, we would not hypothesise many individuals to have two variants only detectable by LR-NGS, however, we recognise that this does represent an avenue for further investigation. As LR-NGS costs continue to fall and bioinformatic pipelines mature we anticipate that LR-NGS will become a first-line test for evaluating genes in which there is high clinical suspicion for a missed variant, but which are difficult to evaluate using short-read approaches, such as *HYDIN*.

In conclusion, *HYDIN* presents a challenge for current SR-NGS and Sanger sequencing due to the presence of *HYDIN2*, and pathogenic variants in *HYDIN* are likely often missed. Moreover, variants in *HYDIN2* may be incorrectly assessed as being present in *HYDIN*. Although bioinformatic masking of *HYDIN2* after SR-NGS reduces the effect of this homology and allows for an increased rate of genetic diagnoses in select cohorts, LR-NGS can overcome all of the challenges presented by this large homology region [32].The proportion of *HYDIN* variants being causative of PCD may vary in different ethnic groups, however we propose that due to the difficulty in identifying pathogenic *HYDIN* variants, *HYDIN* may be an underrepresented cause of PCD in most cohorts. Thus, we feel there are clear benefits of LR-NGS in unsolved cases with a strong clinical phenotype, and we provide further support for the future use LR-NGS as a single test in the clinical environment both to increase the diagnostic rate and to reduce the time required to arrive at a genetic diagnosis.

### Supplementary information

Supplementary material

Supplementary Figure 1

## Data Availability

The datasets generated during this study are available upon request from the corresponding. authors. All variants described in this paper have been submitted to ClinVar (submission number SUB14295852).

## Code availability

The datasets generated during this study are available upon request from the corresponding authors.
