# Supplementary material for: Combined approaches, including long-read sequencing, address the diagnostic challenge of HYDIN in primary ciliary dyskinesia
Source: Eur J Hum Genet. 2024 Apr 11;32(9):1074–85. doi: 10.1038/s41431-024-01599-7 (PMC11369241; doi:10.1038/s41431-024-01599-7)
Supplement: Supplementary file 1 — Supplementary material [file 41431_2024_1599_MOESM1_ESM.docx]

**Supplementary material for manuscript**

**Combined approaches, including long-read sequencing, address the diagnostic challenge of *HYDIN* in primary ciliary dyskinesia**

Andrew Fleming^1^, Miranda Galey,^2,3^ Lizi Briggs,^1^ Matthew Edwards,^1^ Claire Hogg,^4,5^ Shibu John S,^1^ Sam Wilkinson,^1^ Ellie Quinn,^1^ Ranjit Rai,^4^ Tom Burgoyne,^4,6^ Andy Rogers,^4^ Mitali P. Patel,^6,7^ Paul Griffin,^4^ Steven Muller,^1^ Siobhan B. Carr,^4,5^ Michael R. Loebinger,^4,5^ Jane S. Lucas, ^8,9^ Anand Shah,^4,10^ Ricardo Jose,^4^ Hannah M. Mitchison,^6,7^ Amelia Shoemark,^4,11^ Danny E. Miller^2,3,12*^, Deborah J. Morris-Rosendahl^1,5*#^

1. Clinical Genetics and Genomics Laboratory, Royal Brompton and Harefield Hospitals, Guy’s and St. Thomas’ NHS Foundation Trust, London, SW3 6NP, UK
2. Division of Genetic Medicine, Department of Pediatrics, University of Washington and Seattle Children’s Hospital, Seattle, WA, WA 98105, USA
3. Department of Laboratory Medicine and Pathology, University of Washington and Seattle Children’s Hospital, Seattle, WA, WA 98105, USA
4. Primary Ciliary Dyskinesia Centre, Royal Brompton and Harefield Clinical Group, Guy’s and St. Thomas’ NHS Foundation Trust, London, SW3 6NP, UK
5. National Heart and Lung Institute, Imperial College London, London, SW3 6LY, UK
6. Genetics and Genomic Medicine Department, University College London, UCL Great Ormond Street Institute of Child Health, London, WC1N 1EH, UK
7. MRC Prion Unit at UCL, Institute of Prion Diseases, UCL, London, W1W 7FF, UK.
8. Primary Ciliary Dyskinesia Centre, University Hospital Southampton NHS Foundation Trust, Southampton, SO16 6YD, UK
9. Clinical and Experimental Sciences Academic Unit, University of Southampton Faculty of Medicine, Southampton, SO16 6YD, UK
10. MRC Centre of Global Infectious Disease Analysis, Department of Infectious Disease Epidemiology, School of Public Health, Imperial College London, London, W2 1PG, UK
11. Respiratory Research Group, Molecular and Cellular Medicine, University of Dundee, Dundee, DD1 9SY, UK
12. Brotman Baty Institute for Precision Medicine, University of Washington, Seattle, WA, WA 98195, USA

*Joint senior authors

^#^To whom correspondence should be addressed

Email: [d.morris-rosendahl@rbht.nhs.uk](mailto:d.morris-rosendahl@rbht.nhs.uk); Tel. (W): +44 20 7351 8412; (M): +44 755 2020308

**Supplementary Material**

**Material and Methods**

**Cilia Diagnostics**

**High-speed video microscopy (HSVM) and transmission electron microscopy (TEM)**

High-speed video microscopy was performed on fresh epithelial strips in a chamber slide at 37°C using a 100x oil immersion objective and Leica upright microscope (DM-LB) with high-speed video camera (Troubleshooter TS-5 Fastec imaging). Ten strips of ciliated epithelium were recorded, including top and side views, and assessed by a diagnostic scientist for beat pattern and frequency as previously described^1^. Samples were subsequently fixed in cacodylate buffered 2.5% glutaraldehyde for transmission electron microscopy (TEM). Electron microscopy was conducted as previously described^2^; briefly, samples were post-fixed with osmium tetroxide, dehydrated through a series of ethanol and propylene oxide dehydration steps, and embedded in araldite. Blocks were sectioned and stained using uranyl acetate (or uranyl acetate replacement stain) and lead citrate and assessed on a Hitachi 7000 or Jeol 1400+ TEM. 300 ciliary cross sections were counted per section and results reported according to the BEAT-PCD TEM consensus guideline^3^. Where reports were issued prior to publication of the guideline these were converted into guideline compliant language. In cases where variants in *HYDIN* were suspected as a cause, advanced TEM techniques were employed to visualise the C2b projection. These included electron tomography^4^ or image averaging via an inhouse developed program (PCD detect)^3^.

**Immunofluorescence for SPEF2**

Samples taken after 2020 were air dried onto slides and stained for SPEF2 by immunofluorescence^5^. Slides were fixed in 4% paraformaldehyde, blocked in milk powder, and permeabilised with Triton before double labelling with anti-acetylated tubulin (mouse monoclonal antibody T7451, Sigma Aldrich, St. Louis, MO) to visualise cilia and anti SPEF2 (Rabbit polyclonal antibody Sigma - Atlas antibodies HPA039606). Alexafluor secondary antibodies (goat anti-mouse 488 and goat anti-rabbit 594 1:000) were used to visualise the proteins. Slides were preserved using ProLong Gold Antifade Mountant containing DAPI and visualised by confocal microscopy (Leica SP5 confocal microscope). Ten cells were assessed per sample, and the co-localisation of SPEF2 protein with acetylated tubulin of the ciliary axoneme was recorded as present or absent.

**Air-liquid interface culture**

Cells were cultured at 37°C with 5% CO_2._ All plasticware used were coated in 10% collagen (0.3 mg/mL, PureCol 5005.B CellSystems, Troisdorf, Germany). Basal cells were expanded using PneumaCult Ex Plus media supplemented with 0.1% hydrocortisone (STEMCELL Technologies, Vancouver, BC, Canada, #05040 and #07925 respectively), 1% penicillin (5000 U/mL)/streptomycin (5000 µg/mL) (Gibco™Thermo Fisher Scientific, MA, USA #15070063) and Primocin® (InvivoGen, Toulouse, France, #ant-pm-1) on 4-well tissue culture plates (Nunc™Thermo Fisher Scientific, MA, USA #176740). Once confluent, cells were passaged onto T25 cm^2^ flasks before being further passed at a cell density of 83.250 per 250 µl to 12-mm Transwell^®^ with 0.4-µm pore polyester membrane inserts (Corning Life Sciences, Corning, NY, USA, #3460). When on inserts, basal cells were fed with PneumaCult Ex Plus media apically and basolaterally until a confluent monolayer of basal cells was observed. Cells were then taken to air–liquid interface (ALI) by apical media removal and replacement of the basolateral media with PneumaCult ALI media supplemented with hydrocortisone (0.5%) and heparin (0.2%) (STEMCELL Technologies, Vancouver, BC, Canada, ALI kit #05001, #07925 and #07980, respectively) and 1% penicillin (5000 U/mL)/streptomycin (5000 µg/mL) (Gibco™Thermo Fisher Scientific, MA, USA #15070063) and Primocin® (InvivoGen, Toulouse, France, #ant-pm-1). Cells were fed every other day and were ready for HSVM analysis at 4 weeks post ALI.

**Genetic Diagnostics**

**Targeted short-read next generation sequencing: bioinformatic analysis**

Sequence data from targeted SR-NGS was analysed using an automated in-house bioinformatics pipeline. Raw sequence data were demultiplexed (allowing zero mismatches) with Illumina's bcl2fastq v2.20.0.422 software, and read quality was assessed using FastQC v0.10.1^6^. The *HYDIN2* region (chr1:146472566-146914294, GRCh38 reference) was programmatically masked using bedtools v2.27.0 maskfasta feature^7^. Low-quality (<20) reads/bases were trimmed using PrinSeq v0.20.4109^8^, and the high-quality reads were mapped to the *HYDIN2* masked GRCh38 reference genome with Burrows-Wheeler Aligner (BWA) v0.7.17^9^. Marking duplicate reads, local realignment around indels and base quality score recalibration processes were done in GATK v4.0.8.1^10^. Alignment summary metrics, callability and coverage reports were calculated using Picard v1.117, Samtools v1.11^11^, bedtools v2.11.2 and in-house R/Perl scripts. A base was considered ‘callable’ if sequenced with minimum read depth = 10×, base quality ≥ 20 and mapping quality ≥ 10. GATKv4.0.8.1 HaplotypeCaller best practice pipeline^12^ was used to call single-nucleotide (SNP) and indel variants. The following filters were applied to exclude low-quality SNP (FS>60, MQ<40, MQRankSum<−12.5, QD<2.0, and ReadPosRankSum<−8) and Indels calls (QD<2.0, ReadPosRankSum <-20, FS>200). Variants were annotated with Ensembl VEP^13^, and sequence data and coverage were viewed manually through the integrative genomics viewer (IGV)^14^.

**Targeted long-read sequencing**

Libraries for sequencing were prepared using the Oxford Nanopore ligation kit (SQK-LSK110) following the manufacturer’s instructions except that the DNA repair step was run for 30 minutes instead of 5 and the ligation reaction was allowed to proceed for 30 minutes instead of 10. Libraries for targeted LRS (T-LRS) were loaded onto a R9.4.1 flow cell on a Nanopore GridION running MinKNOW version 21.10.8. Adaptive sampling was performed using ReadFish to target *HYDIN* (chr16:70300000–71700000), as well as two control regions (*COL1A1*, chr17:50000000–50250000 and *FMR1*, chrX:147800000–148000000) using GRCh38 as the reference (https://pubmed.ncbi.nlm.nih.gov/33257864/). Each library was allowed to run for 48 – 72 hours and repeat libraries were loaded as needed to increase coverage. For those samples that underwent whole-genome sequencing approximately 600 ng of prepared library was loaded onto a R9 PromethION flowcell and run for 24–36 hours before being stopped, washed, and reloaded.

**Targeted variant testing and primer design**

Targeted testing for SNVs and indels identified by both SR-NGS and LRS was performed using bidirectional Sanger sequencing. Primers were designed using the Sigma-Aldrich OligoEvaluator (http://www.oligoevaluator.com/LoginServlet) and SNPcheck v3 tools (<https://genetools.org/SNPCheck/snpcheck.htm>). Where possible, primer design utilised sequence differences between *HYDIN* and *HYDIN2* for specific amplification.

Any potential CNVs identified by SR-NGS and LRS were confirmed by digital droplet PCR (ddPCR) (BioRad, CA, USA). Primers and probes were designed using the PrimerQuest Tool (https://eu.idtdna.com/Primerquest/Home/Index). *RPP30*, a housekeeping gene with no known copy number variation in exon 7, was used as a control, as recommended by the manufacturer. Fluorescent FAM and HEX dual-labelled probes were used to identify differences between the region of interest and exon 7 of *RPP30* according to the manufacturer's instructions.

**Supplementary Table 1.** Average coverage of each target region (FMR1, COL1A1, HYDIN, and HYDIN2) as well as a background, non-targeted region.

| Sample  (target region) | FMR1  (chrX:147800000-148000000) | COL1A1  (chr17:50000000-50250000) | HYDIN  (chr16:70300000–71700000) | HYDIN2  (chr1:146000000-147000000) | Background  (chr16:60000000-61000000) |
| --- | --- | --- | --- | --- | --- |
| RBH-9929 | 12.2 | 22.0 | 20.8 | 20.6 | 3.4 |
| HM-315-Pa | 5.2 | 8.5 | 8.8 | 8.2 | 3.3 |

**Supplemental references**

1. Rubbo B, Shoemark A, Jackson CL, Hirst R, Thompson J, Hayes J, et al. Accuracy of High-Speed Video Analysis to Diagnose Primary Ciliary Dyskinesia. Chest. 2019;155(5):1008-17.

2. Shoemark A, Dixon M, Corrin B, Dewar A. Twenty-year review of quantitative transmission electron microscopy for the diagnosis of primary ciliary dyskinesia. J Clin Pathol. 2012;65(3):267-71.

3. Shoemark A, Boon M, Brochhausen C, Bukowy-Bieryllo Z, De Santi MM, Goggin P, et al. International consensus guideline for reporting transmission electron microscopy results in the diagnosis of primary ciliary dyskinesia (BEAT PCD TEM Criteria). Eur Respir J. 2020;55(4).

4. Olbrich H, Schmidts M, Werner C, Onoufriadis A, Loges NT, Raidt J, et al. Recessive HYDIN mutations cause primary ciliary dyskinesia without randomization of left-right body asymmetry. Am J Hum Genet. 2012;91(4):672-84.

5. Shoemark A, Frost E, Dixon M, Ollosson S, Kilpin K, Patel M, et al. Accuracy of Immunofluorescence in the Diagnosis of Primary Ciliary Dyskinesia. Am J Respir Crit Care Med. 2017;196(1):94-101.

6. Andrews S. FastQC: a quality control tool for high throughput sequence data. Babraham Bioinformatics, Babraham Institute, Cambridge, United Kingdom; 2010.

7. Quinlan AR, Hall IM. BEDTools: a flexible suite of utilities for comparing genomic features. Bioinformatics. 2010;26(6):841-2.

8. Schmieder R, Edwards R. Quality control and preprocessing of metagenomic datasets. Bioinformatics. 2011;27(6):863-4.

9. Li H, Durbin R. Fast and accurate short read alignment with Burrows-Wheeler transform. Bioinformatics. 2009;25(14):1754-60.

10. McKenna A, Hanna M, Banks E, Sivachenko A, Cibulskis K, Kernytsky A, et al. The Genome Analysis Toolkit: a MapReduce framework for analyzing next-generation DNA sequencing data. Genome Res. 2010;20(9):1297-303.

11. Li H, Handsaker B, Wysoker A, Fennell T, Ruan J, Homer N, et al. The Sequence Alignment/Map format and SAMtools. Bioinformatics. 2009;25(16):2078-9.

12. Van der Auwera GA, Carneiro MO, Hartl C, Poplin R, Del Angel G, Levy-Moonshine A, et al. From FastQ data to high confidence variant calls: the Genome Analysis Toolkit best practices pipeline. Curr Protoc Bioinformatics. 2013;43(1110):11 0 1- 0 33.

13. McLaren W, Gil L, Hunt SE, Riat HS, Ritchie GR, Thormann A, et al. The Ensembl Variant Effect Predictor. Genome Biol. 2016;17(1):122.

14. Robinson JT, Thorvaldsdottir H, Winckler W, Guttman M, Lander ES, Getz G, et al. Integrative genomics viewer. Nat Biotechnol. 2011;29(1):24-6.
