# Supplementary material for: Combined approaches, including long-read sequencing, address the diagnostic challenge of HYDIN in primary ciliary dyskinesia
Source: Eur J Hum Genet. 2024 Apr 11;32(9):1074–85. doi: 10.1038/s41431-024-01599-7 (PMC11369241; doi:10.1038/s41431-024-01599-7)
Supplement: Supplementary file 2 — Supplementary Figure 1 [file 41431_2024_1599_MOESM2_ESM.pptx]

## Slide 1
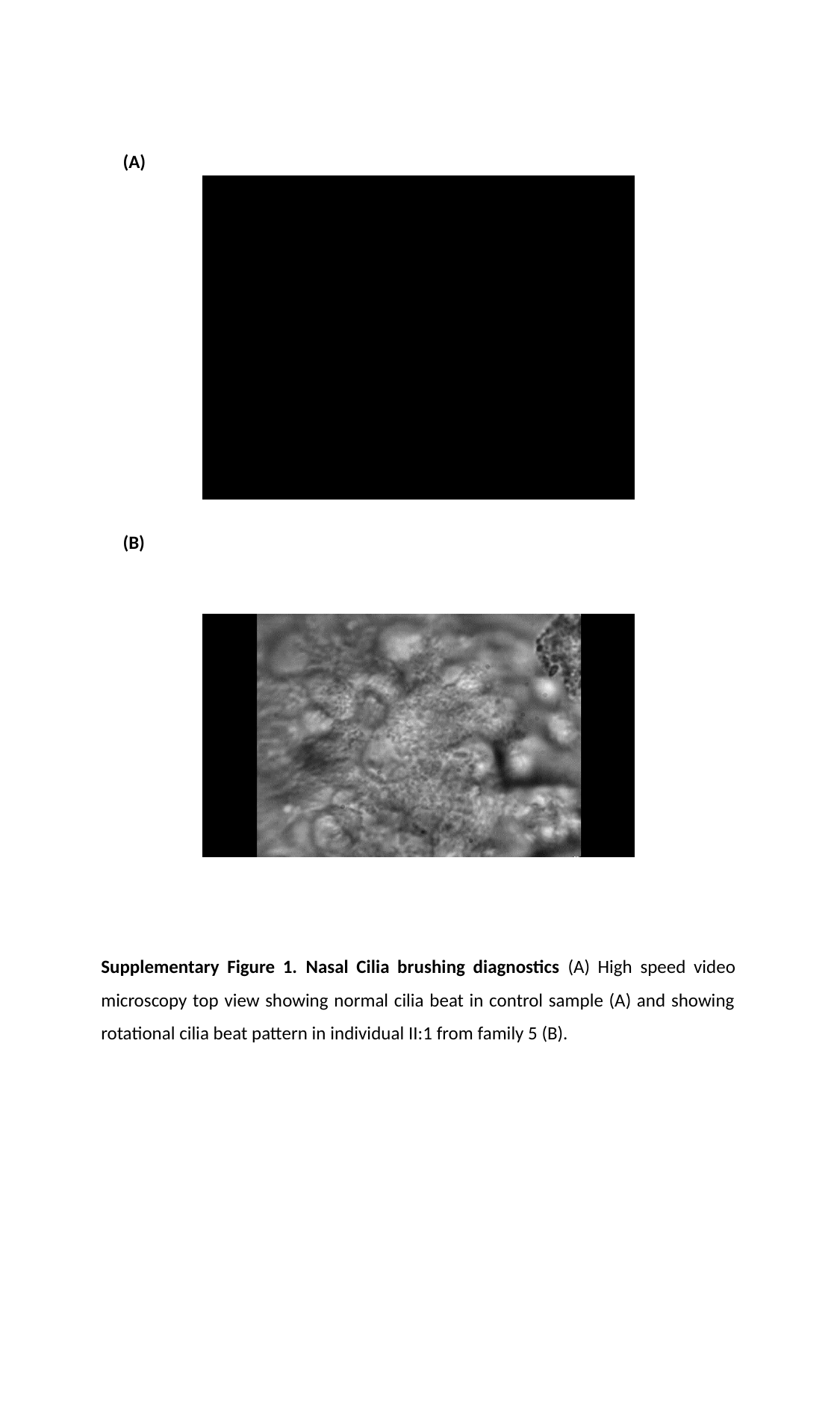

(A)
(B)
Supplementary Figure 1. Nasal Cilia brushing diagnostics (A) High speed video microscopy top view showing normal cilia beat in control sample (A) and showing rotational cilia beat pattern in individual II:1 from family 5 (B).
